# Digital image processing to detect subtle motion in stony coral

**DOI:** 10.1038/s41598-021-85800-7

**Published:** 2021-04-08

**Authors:** Shuaifeng Li, Liza M. Roger, Lokender Kumar, Nastassja A. Lewinski, Judith Klein-Seetharaman, Alex Gagnon, Hollie M. Putnam, Jinkyu Yang

**Affiliations:** 1 Department of Aeronautics and Astronautics, University of Washington, Seattle, WA 98195-2400 USA; 2 Department of Chemical and Life Science and Engineering, Virginia Commonwealth University, Richmond, VA USA; 3 Department of Physics, Colorado School of Mines, Golden, CO USA; 4 Department of Chemistry, Colorado School of Mines, Golden, CO USA; 5 School of Oceanography, University of Washington, Seattle, WA USA; 6 Department of Biological Sciences, University of Rhode Island, Kingston, RI 02881 USA

**Keywords:** Image processing, Marine biology

## Abstract

Coral reef ecosystems support significant biological activities and harbor huge diversity, but they are facing a severe crisis driven by anthropogenic activities and climate change. An important behavioral trait of the coral holobiont is coral motion, which may play an essential role in feeding, competition, reproduction, and thus survival and fitness. Therefore, characterizing coral behavior through motion analysis will aid our understanding of basic biological and physical coral functions. However, tissue motion in the stony scleractinian corals that contribute most to coral reef construction are subtle and may be imperceptible to both the human eye and commonly used imaging techniques. Here we propose and apply a systematic approach to quantify and visualize subtle coral motion across a series of light and dark cycles in the scleractinian coral *Montipora capricornis*. We use digital image correlation and optical flow techniques to quantify and characterize minute coral motions under different light conditions. In addition, as a visualization tool, motion magnification algorithm magnifies coral motions in different frequencies, which explicitly displays the distinctive dynamic modes of coral movement. Specifically, our assessment of displacement, strain, optical flow, and mode shape quantify coral motion under different light conditions, and they all show that *M. capricornis* exhibits more active motions at night compared to day. Our approach provides an unprecedented insight into micro-scale coral movement and behavior through macro-scale digital imaging, thus offering a useful empirical toolset for the coral research community.

## Introduction

Reef-building corals, as keystone organisms, support diverse marine communities and provide a host of ecosystem functions for associated creatures, such as macrofauna. They are composed of coral organisms living in symbiosis with photosynthetic dinoflagellate algae and a complex of additional bacterial, archaeal and fungal communities^1^. Coral behavior, physiology and ecology are impacted by anthropogenic global climate change through the global rise in sea-surface temperatures and ocean acidification^2^. Simultaneously, coral reefs have experienced substantial decline due to disease outbreaks^3^, overfishing^4^, coastal development and associated runoff^5^. The increasing frequency of marine heat waves has also resulted in mass coral mortality^6,7^. The combination of these stressors is threatening coral reefs at an unprecedented scale.

One notable aspect of corals is their dynamic motion (i.e., temporal changes of the polyp and tissue behavior), since it could play an important role in coral physiology and the coral health in a changing environment. Thus, understanding coral motion will help us to better assess coral performance in a proactive manner and understand coral physiology and the coral health in a changing environment in terms of the light condition, temperature, pH and other environmental variables^8–15^. Some soft corals, such as the family of *Xeniidae*, exhibit a unique, rhythmic pulsation, which is believed to enhance photosynthesis^16,17^. However, compared with the soft corals, the scleractinian corals responsible for the framework structure of most reefs mineralize a rock-like skeleton made of calcium carbonate. In many species of scleractinian corals, the motion of tissue is more subtle or imperceptible. Therefore, visualizing and quantifying the dynamic motion in order to extract information on coral behavior remains challenging for researchers studying scleractinia.

The dynamic motion of scleractinian corals interacts with a number of physiological processes and environmental responses. For example, some branched corals with small polyps will expand their tentacles to expose the photosynthetic symbionts to light during daytime^15,18,19^. However, under the strong light, the coral will retract the tentacles to protect photosynthetic symbionts from irradiance^15,18,19^. These behavioral differences indicate how coral movement can both impact and act as an indicator of ecological and physiological tradeoffs. Besides, some tentacles also serve as the probes to detect and kill competitors that settle within the wide aggressive reach of these massive corals^20^. The ciliary motion of tentacles also contributes to the mass transfer^21–23^. In areas away from coral polyps and their tentacles, the coenosarc, more subtle tissue movements have been revealed. These waves of tissue movement may speculatively be involved in enveloping or pumping seawater to different reservoirs within a coral^24^. Recently, seawater exchange rates in a growing coral were found to respond to stressful conditions like extreme ocean acidification^25^. Taken together, experiments like these hint at the rich connections that may exist between tissue movement, physiology, and how corals respond to environmental changes. New techniques that can image and quantify subtle tissue movements could uncover the role of motion in coral health and help predict the fate of coral reefs in a changing ocean.

Imaging is a powerful way to provide information about the time-varying nature of the world. Photogrammetry microscopic imaging, and time-lapse imaging have resulted in the capacity of detecting change at both reef^26,27^ and cellular levels^28–30^. New imaging techniques borrowed from other fields, such as mechanics, aerospace engineering and biological engineering, promise even more detailed and quantitative information that could be applied to address the fine-scale analysis of coral movement under a changing environment^31,32^. For example, correlation-based image registration and tracking methods such as digital image correlation (DIC) and particle image velocimetry (PIV) measure the mechanics of materials and fluids^31,33^, and thus could be used to map movement of the coral tissue surface^22^. Optical flow is another effective method to demonstrate the movement between the camera and moving objects^32^. These approaches can potentially quantify the pixel-level motion in terms of displacement and velocity in biological fields.

To provide the evident motions, previous attempts on magnifying subtle and imperceptible movements have been made along two perspectives: *Lagrangian* perspective and *Eulerian* perspective^34–37^. For instance, the imperceptible change in face color can be magnified to estimate the heart rate^38^, and the functional role of tectorial membrane in mammalian hearing can be revealed^39^. These approaches have rarely been used in marine biological systems including corals where they could quantify and visualize motion and further build a bridge between imaging techniques and biomechanics.

Here, we select one species of hard coral, *Montipora capricornis*, to study the dynamic motion of coral polyps and coenosarc under diel cycling light conditions using time lapse imaging. We first quantify the coral motion by DIC and extract barely visible mechanical quantities in terms of displacements and strains. Next we present the optical flow to show the velocity field and motion polarization. These two methods offer physiological information in corals with high consistency, which could not have been sensed by naked eyes. Typical modes of motion are visualized by the phase-based motion magnification, which clearly shows a pattern of motion related to the corals’ sensitivity to the changing light conditions. We provide a systematic and quantitative approach for characterizing and analyzing subtle and/or imperceptible coral tissue movements, thereby opening a new vista in coral behavioral, physiological, and cellular analysis.

## Results

### Experimental observation of Montipora capricornis

**Figure 1:**
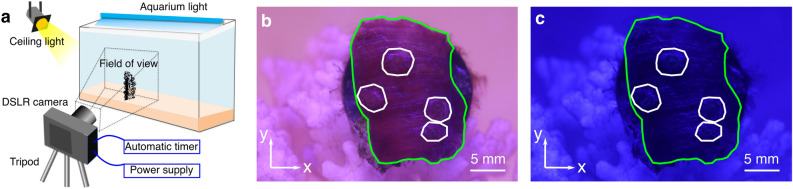
Experimental observation of *Montipora capricornis.* (**a**) Schematic of experimental setup of a DSLR camera, aimed at the corals in the aquarium. (**b**) Daylight pictures were taken under white light, while (**c**) nighttime pictures were taken under blue light. The green curves in (**b**) and (**c**) enclose the coral tissue surface. The white curves in (**b**) and (**c**) enclose the polyps. Scale bars are shown in (**b**) and (**c**).

Figure 1a shows a schematic of the experimental setup used to observe the coral *M. capricornis*. The planar structure and textures on the surface of *M. capricornis* are beneficial to the implementation of our algorithm, which makes this species a good candidate for this proof of concept study. It includes a digital single lens reflex (DSLR) camera with a macro lens to take pictures of the fine structures of coral tissue surface. To study the effect of light on coral tissue motion, aquarium light and ceiling light were used to mimic light conditions during the day and the night. Two hundred pictures are taken for each light condition with a rate of 30 frames per hour. Experimental setup details are shown in Supplementary Fig. 1 and are described in the Methods section.

Figure 1b,c display the pictures taken at day and at night, respectively. The green curves represent the coral tissue margins. The white curves indicate the positions of polyps. Four polyps with short tentacles are on the coral surface. The nearly planar arrangement of the coral provided the opportunity to obtain motion information from all polyps on the coral’s surface. The heterogeneity of the tissue on surface creates texture that is sufficient for applying DIC and optical flow techniques. Luminance (as calculated and shown in Supplementary note 1 and Supplementary Fig. 2) was smaller in the blue light at night than at day. Besides, the hue is more blueish at night due to the lack of the ceiling light. Visualization of coral dynamic motion, not perceptible by the human eye, can be observed from the time-lapse video played at 10 frames per second (× 1200 speed; Supplementary movie 1).

**Figure 2:**
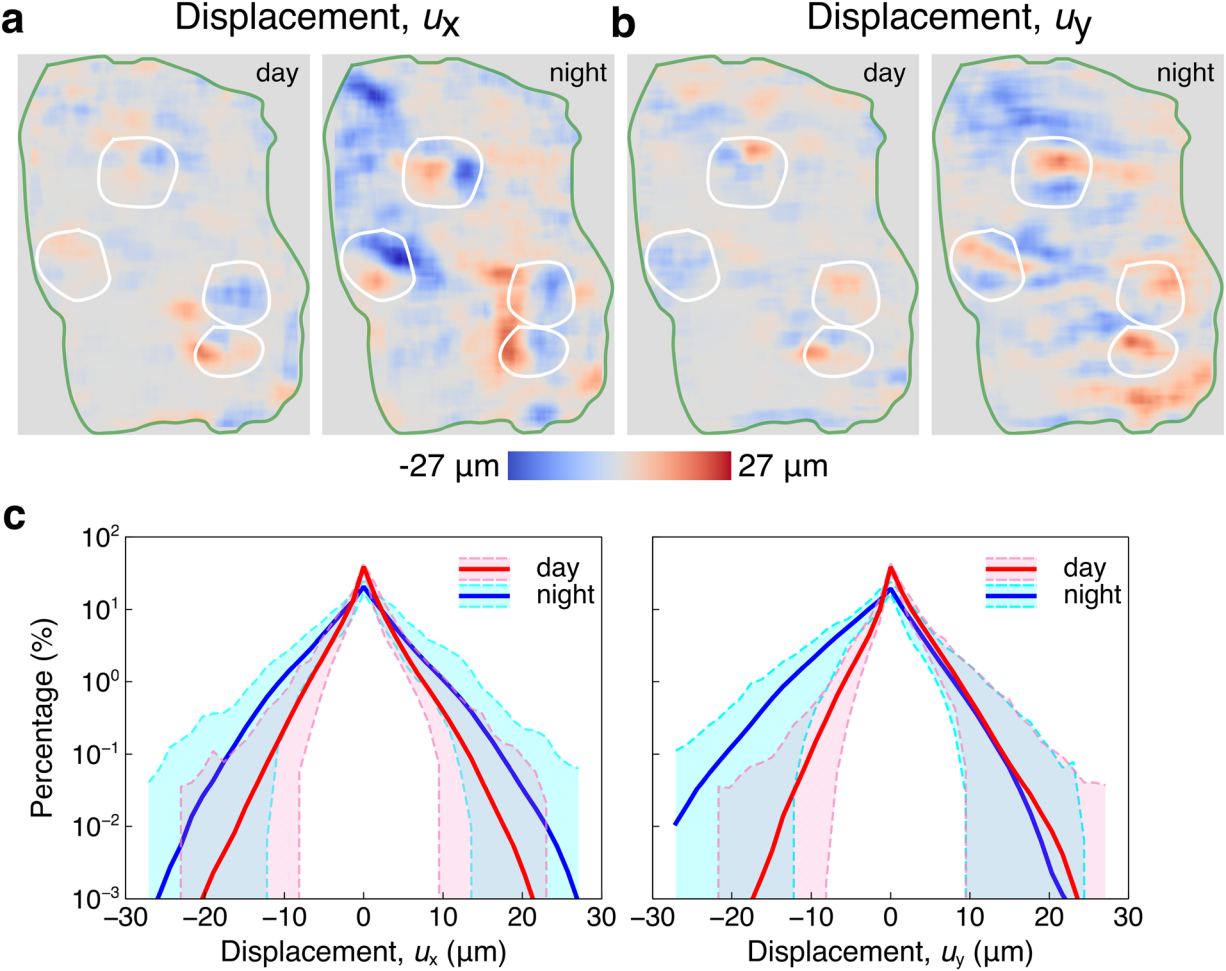
Displacements of the coral tissue surface from digital image correlation. (**a**) The displacements $u_{x}$ along horizontal direction between the reference picture and the second picture at day and night are shown in the left and right panel, respectively. (**b**) The displacement $u_{y}$ along vertical direction between the reference picture and the second picture at day and night are shown in the left and right panel, respectively. Green curves in (**a**) and (**b**) represents the edge of the fragment and white curves enclose the polyps. (**c**) The percentage histograms of displacement $u_{x}$ and displacement $u_{y}$ are shown in the left and right panels, respectively. The red and blue curves indicate the means of each displacement during day and night. The pink and cyan regions are variations during day and night, respectively.

### Characterization by digital image correlation

In order to quantify coral tissue motion from a biomechanics perspective, we used a DIC technique to characterize the deformation of the surface relative to an initial reference picture (the first picture). Displacements ($u_{x}$) along the horizontal direction between the reference picture and the second picture at day and night are shown in Fig. 2a, respectively. The strongest motions are concentrated around polyps and the edge of the fragment. At night, most of the coral tissues surface undergoes horizontal motion, while few coral tissues are moving horizontally at day. Displacements ($u_{y}$) along the vertical direction were also quantified between the reference picture and the second picture at day and night, respectively (Fig. 2b). Similar to the distribution of displacement $u_{x}$, the strongest motions are concentrated around polyps and the edge of the fragment. Vertical tissue motion is more widespread across the coral at night compared to daytime. The displacement of both $u_{x}$ and $u_{y}$, in comparison to the reference picture between day and night, can be seen in videos created from two hundred pictures (Supplementary movie 2 and Supplementary movie 3). These visualizations highlight that the coral tissue surface is moving dynamically over time, with more movement observed during the night than during the day.

In order to explore the statistics of DIC results, we show the histogram of displacements $u_{x}$ and $u_{y}$. Across ~ 13 h, histograms of displacements $u_{x}$ and $u_{y}$ are continuously changing (Supplementary movie 4). Figure 2c exhibits the percentage histograms of the displacement $u_{x}$ and $u_{y}$ during day and night in the left and right panels. The red and blue curves in each panel indicate the mean of displacement distribution during day and night. The corresponding variation ranges are shown in pink and cyan. According to the histogram (Fig. 2c), most of the areas of the coral tissue surface are barely moving, i.e., nearly zero displacement. For the moving part, more areas of the coral tissue surface are contributing to the motion at night since the blue curves are above the red curves, coinciding with the displacements shown in Fig. 2a,b. Furthermore, the histogram of displacement $u_{x}$ (Fig. 2c) illustrates the nearly symmetric distribution, suggesting that there is no motion preference and the system is balanced along the horizontal direction. However, the histogram of displacement $u_{y}$ shows an asymmetric distribution. During the day, the coral tissue surface tends to move upwards because there are more positive values, while at night, the tissue tends to move downwards, which indicates that motion preference based on the light condition is shown along the vertical direction.

**Figure 3:**
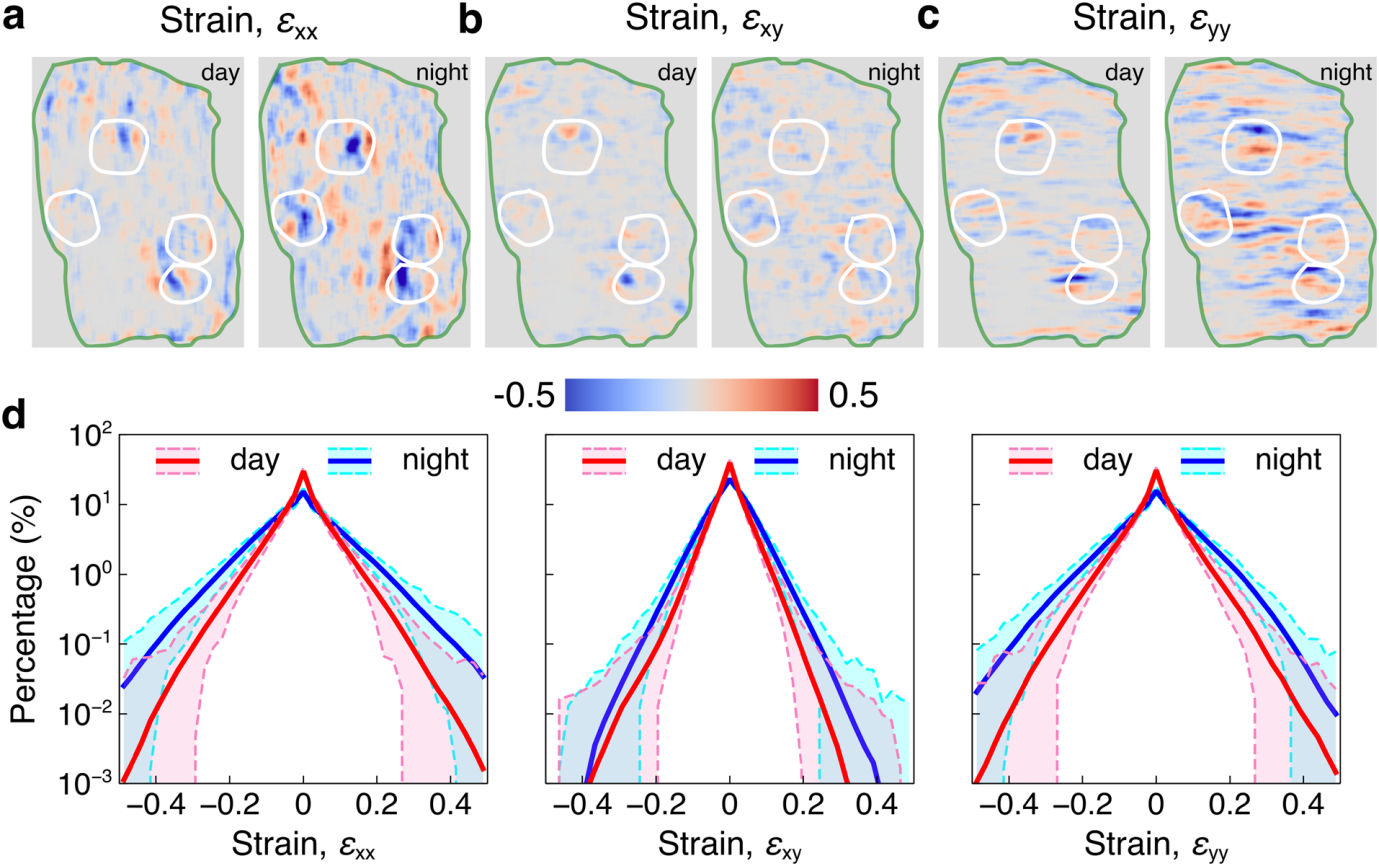
Strains of the coral tissue surface from digital image correlation. **(a–c**) Strains $\varepsilon_{xx}$, $\varepsilon_{xy}$ and $\varepsilon_{yy}$ between the first and the second picture from day and night are shown in the left and right panel, respectively. Green curves in (**a**) and (**b**) represents the edge of the fragment and white curves enclose the polyps. (**d**) The percentage histogram of the strains $\varepsilon_{xx}$, $\varepsilon_{xy}$ and $\varepsilon_{yy}$ are shown in the left, middle and right panels, respectively. The red and blue curves indicate the means of each strain during day and night. The pink and cyan regions are variations during day and night.

Strain measures the deformation of the material to identify whether it is under tension or compression. The linear strain is considered here. To obtain the accurate strain, smoothing and noise reduction of displacement field are necessary. We adopt the point-wise local least-square fitting technique^40,41^. The strain calculation window is set to 19 $\times$ 19 points, which provides a trade-off between accuracy and smoothness of strain estimation. Supplementary movie 5 shows the normal strain $\varepsilon_{xx}$ and $\varepsilon_{yy}$, shear strain $\varepsilon_{xy}$ during day and night, respectively. The strains between the second picture and the reference picture are shown in Fig. 3a–c. Figure 3a shows the normal strain $\varepsilon_{xx}$ during day and night. The largest strain is concentrated around the polyps and obviously the distribution of $\varepsilon_{xx}$ is more evident at night (Fig. 3a), suggesting that more of the coral tissue surface participate in the motion at that time. Figure 3b,c, indicating shear strain $\varepsilon_{xy}$ and normal strain $\varepsilon_{yy}$, show the same features as Fig. 3a. Compared with the $\varepsilon_{xx}$, $\varepsilon_{yy}$ has clearer alignment along the wrinkles on the coral surface, indicating the anisotropy of the coral tissue surface motion. Similar to the analysis of displacement, the corresponding histograms are displayed from left to right in Fig. 3d. The red and blue curves in each panel indicate the mean of strain distribution during day and night throughout the strain result. The corresponding variation ranges are shown in pink and cyan. Most of the coral tissue was not either under tension or compression due to the nearly zero strains, which is corresponding to the nearly zero displacements in the above analysis. Corresponding to the displacement, strain measurements also show larger values at night.

We note that we have also conducted a noise effect study to test the statistical significance of the DIC results presented above. Although correlation functions act to normalize images during image processing, quantification of noise effects provide a further verification of the observed patterns^42^. The enclosed coral skeleton on which our coral is fixed, shown in Supplementary Fig. 3, is analyzed by DIC. Supplementary Fig. 4 and Supplementary Fig. 5 show the displacements and strains information of the coral skeleton. Details and analysis are in the Supplementary note 2. The results indicate that the DIC results are valid, since the noise is smaller than the extracted signal (Supplementary Fig. 4 and Supplementary Fig. 5; see also Supplementary note 2 and Supplementary movie 6).

**Figure 4:**
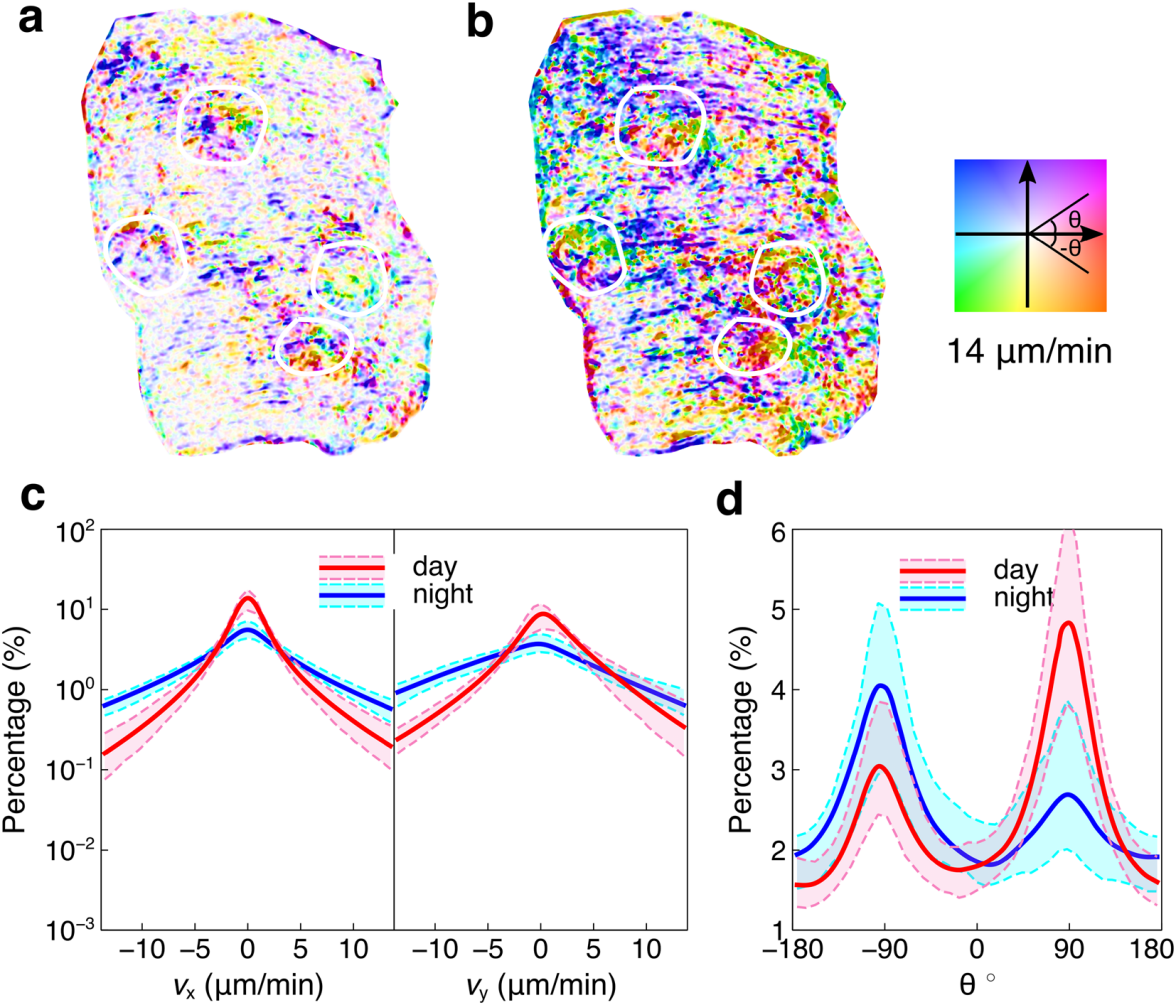
Optical flow of the coral tissue surface. (**a**,**b**) Optical flow between the second and first pictures from day and night, respectively. White circles mark the position of polyps. (**c**) The percentage histograms of velocities $v_{x}$ and $v_{y}$ along horizontal and vertical directions. The red and blue curves indicate the means of each velocity from day and night. The pink and cyan regions are variations between day and night. (**d**) The percentage histogram of direction of velocity. The red and blue curves indicate the means of each angle from day and night. The pink and cyan regions are variations during day and night.

**Figure 5:**
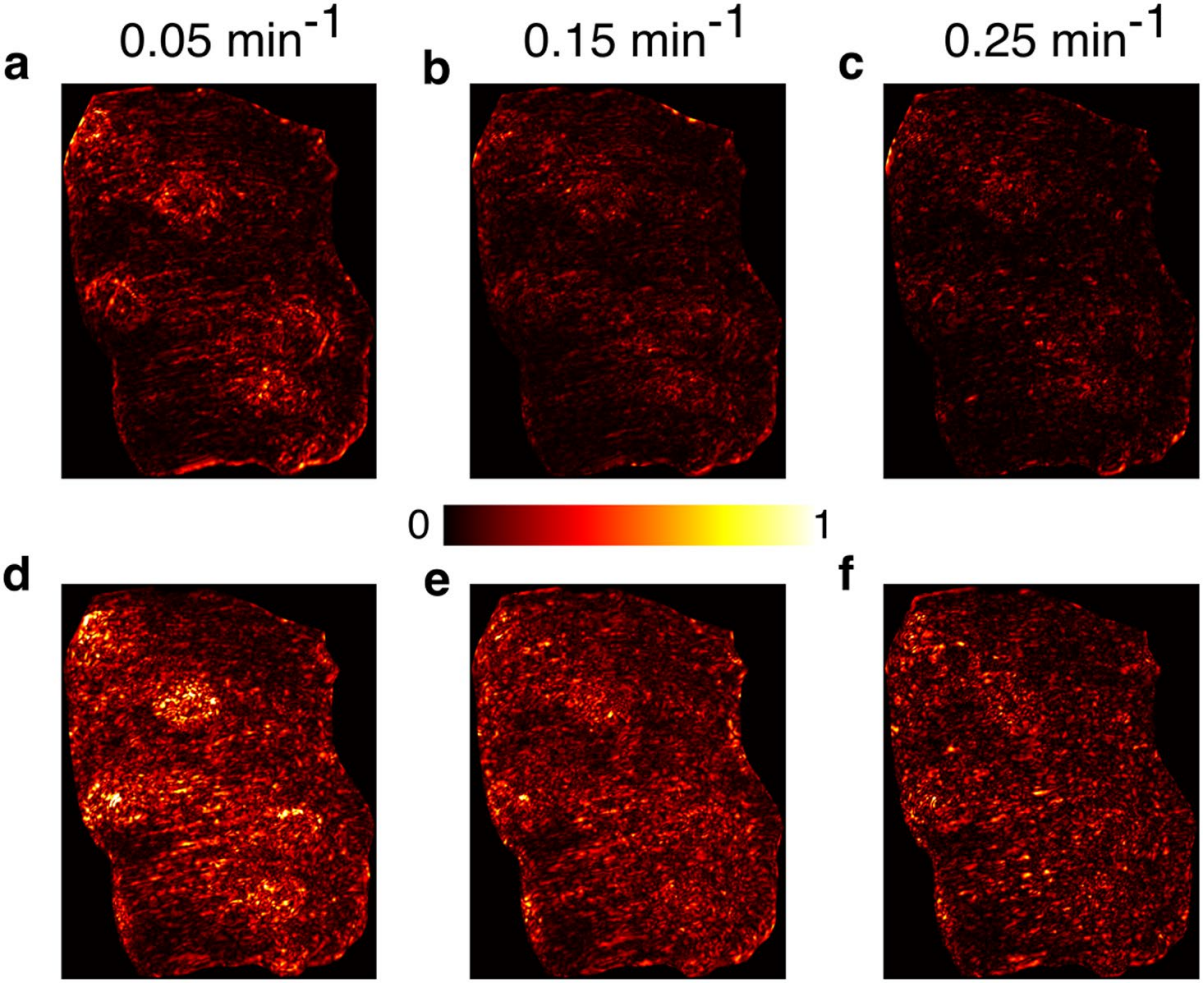
Mode shape of coral tissue motion during day and night. (**a–c**) Mode shapes of coral tissue motion during day at frequencies 0.05 min^−1^, 0.15 min^−1^ and 0.25 min^−1^, respectively. (**d–f**) Mode shapes of coral tissue motion during night at frequencies 0.05 min^−1^, 0.15 min^−11^ and 0.25 min^−1^, respectively.

### Characterization by optical flow

Measuring optical flow is an effective method to explore the pixel-wise motion information such as velocity. In order to increase the signal-to-noise ratio, we assume the motion field is constant in a small window (21 × 21) around each pixel and the smoothing kernel (19 × 19) is used for reducing noise. The basic optical flow equation is solved for all pixels in the window by the least squares criterion, which is also known as the Lucas-Kanade method^43^. Similar to DIC, for each case, we take the first picture as the reference and calculate the optical flow for two hundred pictures. Supplementary movie 7 shows the velocities of the optical flow during day and night. Figure 4a,b exhibit the optical flow between the second picture and the reference picture. The optical flow is encoded by the color square. The color indicates the direction of velocity and the saturation of color represents the magnitude of velocity. The colors with high saturation are around the polyps (marked by black crosses) and margins, and colors with low saturation are distributed around the coral tissue surface margin. At night, there are more colors with high saturation in the optical flow, suggesting that coral movement is higher in magnitude at night.

The histograms of velocity $v_{x}$ and $v_{y}$ along horizontal and vertical directions are studied to explore the statistics of optical flow. Figure 4c shows the histogram of $v_{x}$ and $v_{y}$. The red and blue curves in each panel indicate the mean of corresponding velocity distribution from day and night. The corresponding variation ranges are shown in pink and cyan dashed lines. While the DIC extracts the displacement information, optical flow can provide pixel-wise velocity information. Similar to DIC results, along the horizontal direction, the histogram is symmetric (Fig. 4c), indicating that the coral tissue surface does not have statistically significant motion preference along the horizontal direction. The histogram of $v_{y}$ shows the similar trend with the displacement $u_{y}$ from DIC (Fig. 4c). During the day, the coral tissue surface tends to present a positive velocity translating upwards motion, while the tissue tends to move downwards with a negative velocity at night. This observation is even more evident in Fig. 4d, which offers a histogram of direction of optical flow. 90° and – 90° represent the upward and downward direction, respectively. According to Fig. 4d, the dominated motion is along the vertical direction whatever light condition the coral is under since there are evident peaks in 90° and –90°. Besides, in different light conditions, the motion directions are different. At day, there is a higher peak in 90°, which means that the tissue tends to move upwards, while at night, the higher peak in –90° suggests the downwards motion. Similarly, we conducted the noise effect study as shown in Supplementary Fig. 6. The results show that the velocities on the coral skeleton are smaller, thus supporting the validation of the analysis mentioned above. Details are described in Supplementary note 3.

### Motion microscope for the coral tissue surface

Exploring the biological and physical modes of coral tissue motion is crucial to understand the coral behaviors. Here we further process the optical flow result to obtain the deformation of coral tissue surface at different frequencies and magnify the biological modes of this movement to make them visible to human eyes. To this end, we perform the Fourier transform on the optical flow results and extract the absolute value of velocity in different frequencies. Zero padding is used to increase the frequency resolution. Supplementary movie 8 shows mode shapes of coral tissue in the frequency range of 0 min^−1^ to 0.25 min^−1^ (Nyquist frequency). Typical mode shapes from day and night are shown in Fig. 5a–f, respectively, after normalization. As the frequency increases, the intensity of the motion decreases. In the frequency 0.05 min^−1^ during the day, as shown in Fig. 5a, the motion is concentrated around the polyps and margins. In stark contrast, Fig. 5d exhibits the mode shape in the frequency 0.05 min^−1^ where the motion is not only around the polyps and margins, but also on the coral tissue surface. Under both light conditions, a greater proportion of coral tissue contributes to the motion with the increasing frequency. Overall, the mode of extremely slow coral tissue motion is observed, which coincides with the phenomenon observed in DIC and optical flow.

In order to visualize the mode shape in different frequencies, we adopt the phase-based motion magnification^16^. The complex steerable pyramid, which is an over-complete transformation that decomposes an image according to spatial scale, orientation and position, is used to magnify the motion in a specific frequency range^44^. The image is completely decomposed into amplitudes $A_{\omega ,\theta } \left( {x,y} \right)$ and phases $\varphi_{\omega ,\theta } \left( {x,y} \right)$ at every scale $\omega$ and orientation $\theta$. We augment the motion 75 times by magnifying phase in the frequency range of interest. We choose three frequency ranges, 0.04 min^−1^–0.06 min^−1^, 0.14 min^−1^–0.16 min^−11^ and 0.22 min^−1^–0.24 min^−1^ to demonstrate the motion microscope for coral tissue mode shapes. Supplementary movie 9 shows the magnified video played at 10 frames per second, which is made of pictures taken during the day. The movie presents the polyps motion in the low frequency range and the coral tissue surface motion in the high frequency range. Besides, the motion magnitude is decreasing as the frequency increases. Supplementary movie 10 shows the magnified video at night in the frequency range mentioned above. According to the video, more coral tissue is involved in the motion, which coincides with the mode shape analysis from the optical flow.

## Conclusion

We applied digital image correlation, optical flow, and motion microscopy techniques to a series of images of *M. capricornis*. Though this hard coral has slow and subtle tissue motion, which is hard to capture not only with naked eyes, but also with optical apparatus, we have successfully extracted its responsiveness to light conditions based on ~ 13 h of sampled digital images. The blending of powerful and effective tools from mechanics and computer science and coral biology opens avenues for studying coral physiology from macro-scale pictures. It is more than likely that different coral species present different levels of tissue motion in direct relation with their overall morphology (e.g. branching, corymbose, digitate, encrusting, foliose, laminar, massive, submissive, solitary, tabulate). With this consideration in mind, imaging surface tissue motion could help predict species-specific responses to sediment smothering^45,46^ and whether corals adapt the movements of the surface tissue according to particle loading in the water column in relation to flood plumes and land runoff. Reef-scale responses to flood plumes triggered by severe weather events could then be predicted according to coral surface tissue motion measurements, depending on coral cover and species representation. Investigations linked to coral tissue motion should also be considered with respect to exposure to pollutants generated by anthropogenic activities, e.g., oil and chemical dispersant^47,48^. This systematic approach offers us possibilities to quantify and visualize the subtle coral tissue surface motion and important physical and biological modes in a time-efficient, yet accurate manner. In this proof of concept study, we only focus on the effect of light to motion for a single coral, but the proposed approach can be generalized to study the effect of a variety of environmental variables on coral tissue motion. Furthermore, this method could be applied to investigate the links between tissue motion and coral mucus (production, thickness, composition, spread), especially in response to excess sediment or bacterial infection^49,50^.

## Method

### Aquarium maintenance

The *M. capricornis* (around 15 mm × 20 mm) was bought from a local store (Seattle Corals Aquariums) and was raised in a 11.36 L aquarium with specific density = 1.024 g/cm^3^ and pH = 8.4. Artificial seawater was made from Instant Ocean Reef Crystals Reef Salt with the salinity = 34.1 ppt. A one third water change was carried out every 3 days to maintain steady aquarium conditions. Regular tests on pH, ${\text{NH}}_{4}^{ + }$,${\text{NO}}_{2}^{ - }$ and ${\text{NO}}_{3}^{ - }$ were conducted to ensure suitable water quality for coral growth. The continuous water flow within the tank was provided by the Hydor Koralia Nano Aquarium Circulation Pump with 908.5 L per hour flow rate. The temperature of the water was controlled by the 50-W FREESEA submersible heater and was maintained at 25.5 °C. Light was provided on a 14.5:9.5 light: dark cycle using a 6-W NICREW ClassicLED aquarium light and the ceiling light in the laboratory. The aquarium light emits blue light with 380 lumens, and it was turned on all day, while the ceiling light emits white light and it was controlled manually to be turned on at day and turned off at night.

### Pictures acquisition

We used a Canon EOS 5D Mark IV and EF 100 mm f/2.8L Macro IS USM and timer to take pictures for the *M. capricornis* every 2 min automatically. The parameters for the camera were: F11, ISO4000, 1/10 s. The camera was put on a tripod to make it stable and ready for the long-period shooting. The macro lens was perpendicular to the wall of the aquarium to avoid the blurred effect caused by the refraction of light. The distance between the macro lens and the wall of the aquarium was 12.5 cm.

## Supplementary Information

Supplementary Video 1.

Supplementary Video 2.

Supplementary Video 3.

Supplementary Video 4.

Supplementary Video 5.

Supplementary Video 6.

Supplementary Video 7.

Supplementary Video 8.

Supplementary Video 9.

Supplementary Video 10.

Supplementary Information 1.

## Data Availability

Data and Matlab codes for digital image correlation, optical flow, and motion microscopy are available for download in Open Science Framework. https://doi.org/10.17605/OSF.IO/49KMH.
