# Supplementary material for: Digital image processing to detect subtle motion in stony coral
Source: Sci Rep. 2021 Apr 8;11:7722. doi: 10.1038/s41598-021-85800-7 (PMC8032694; doi:10.1038/s41598-021-85800-7)
Supplement: Supplementary file 11 — Supplementary Information 1. [file 41598_2021_85800_MOESM11_ESM.docx]

Supplementary Information

Digital image processing to detect subtle motion in stony coral

Shuaifeng Li^1^, Liza M. Roger^2^, Lokander Kumar^3^, Nastassja Lewinski^2^, Judith Klein-Seetharaman^4^, Alex Gagnon^5^, Hollie M. Putnam^6^, Jinkyu Yang^1^

^1^Department of Aeronautics and Astronautics, University of Washington, Seattle, Washington, 98195-2400, USA

^2^Chemical and Life Science and Engineering, Virginia Commonwealth University, Richmond, VA, United States

^3^Department of Physics, Colorado School of Mines, CO, USA

^4^Department of Chemistry, Colorado School of Mines, CO, USA

^5^School of Oceanography, University of Washington, Seattle, WA, USA

^6^Department of Biological Sciences, University of Rhode Island, Kingston, RI, 02881, USA

**Supplementary note**

Supplementary note 1

**Extraction of luminance of images.** In our algorithm, we use the luminance of the image to represent the light intensity. Our original images in RGB color space are converted to NTSC color space with three components: Y, I and Q, using the following equation:

$$\left[ \begin{matrix} Y \\ I \\ Q \end{matrix} \right]=\left[ \begin{matrix} 0.299 & 0.587 & 0.114 \\ 0.596 & -0.274 & -0.322 \\ 0.211 & -0.523 & 0.312 \end{matrix} \right]\left[ \begin{matrix} R \\ G \\ B \end{matrix} \right]$$

where Y component represents luminance or brightness of the image in the range from 0 to 1. The other components carry the hue and saturation information. We define the mean of the Y component throughout the image as the luminance of this image. Supplementary Figure 2 shows the luminance of images taken at day and at night. The luminance at day is around 0.48 while the luminance at night is around 0.16, which coincides with the intuition that the luminance at day is larger.

Supplementary note 2

**Digital image correlation of the coral skeleton.** The coral skeleton is in the aquarium which is indicated by the yellow curves in Supplementary figure. 3a and Supplementary figure. 3b. The *Montipora capricornis* is fixed on a coral skeleton. The coral skeleton is not supposed to develop notable motions under the water flow or other factors, which provides a good opportunity to test the validation of DIC and optical flow. The first picture is considered as the reference picture. Supplementary figure 4a shows the displacements $u_{x}$ between the second picture and the reference picture at day and at night from left to right. In comparison with the displacement of coral shown in Fig. 2a, the results of coral skeleton at day and at night do not show obvious distinction but are smaller than the results of coral. We also investigate the displacements $u_{y}$ at day and at night shown in Supplementary figure 4b. No obvious difference is observed from the results. Similarly, $u_{y}$ is smaller than the results of coral. The histograms of displacements $u_{x}$ and $u_{y}$ are presented in Supplementary figure 4c. The red and blue curves in each panel indicate the mean of displacement distribution at day and at night throughout the displacement result. The corresponding variation ranges are shown in red and cyan dashed lines. The results show that most of the parts are not moving since the displacements are zeros. The means of both displacements $u_{x}$ and $u_{y}$ are either the same or slightly different at day and at night. Besides, the noise is smaller than the results of coral. It suggests that the results are not significantly affected by the light condition, which verify the validation of the digital image correlation algorithm.

The same phenomenon can be found in the strain information. The strains between the second picture and the reference picture are shown in Supplementary figure 5a, 5b and 5c. Supplementary figure 5a and 5c shows the normal strain $\varepsilon_{xx}$ and $\varepsilon_{yy}$ at day and at night, and 5b shows the shear strain $\varepsilon_{xy}$. Similar to the displacements analyzed above, the strains also exhibit no obvious distinctions. The histograms of $\varepsilon_{xx}$, $\varepsilon_{xy}$ and $\varepsilon_{yy}$ shown in Supplementary figure 5d make it clearer. The means of strains at day are in close proximity to the values at night. In general, the comparison of DIC results between coral and coral skeleton supports the validation of DIC algorithm.

Besides, we also analyze the possibility that the difference between day and night is caused by accident. Supplementary movie 6 shows the histograms representing the distributions of displacements when the consecutive picture is considered as the reference picture. From the movie, the displacement at night is larger than that at day. Moreover, positive and negative displacements are shown in the movie, suggesting that the coral is fixed on the coral skeleton during the experiment and only motions of polyps and coral skin are recorded. Another reference picture is also considered. We also take the 20^th^, 40^th^ and 100^th^ picture as the reference picture, respectively, and calculate the histograms of displacements, shown in Supplementary movie 6. They all show that the displacement at night is larger than that at day. Moreover, positive and negative displacements are shown in the movies. The above analysis indicates that our result is obtained statistically rather than by accident.

Supplementary note 3

**Optical flow of the coral skeleton.** In order to verify the validation of optical flow, we also carry out the study of optical flow on coral skeleton enclosed by yellow curves in Supplementary figure 3. Supplementary figure 6a and 6b exhibit the optical flow of the coral skeleton encoded by the color square with the same scale between the second picture and the first picture at day and at night, respectively. Similar to the DIC result, the top right corner is in the low saturation, meaning that it is lack of the coral skeleton. Besides, there is no significant difference between Supplementary figure 6a and 6b from the perspective of color distribution. The histograms shown in Supplementary figure 6c illustrate the distribution of velocities $v_{x}$ and $v_{y}$. More parts are involved in the motion at day, which is on the opposite side of the case of coral. According to the symmetry of the histograms, the coral skeleton tends to have a positive velocity $v_{x}$ and a negative velocity $v_{y}$ whatever light condition the coral skeleton is under. Besides, the values are smaller than the velocities on coral. The histogram of direction is shown in Supplementary figure 6d. Probably because of water flow, the motion direction is along the horizontal direction, which is 0° and 180°. Apart from that, there is no significant peak in the histogram. Overall, the comparison of optical flow results between coral and coral skeleton supports the validation of optical flow algorithm.

**Supplementary figure**


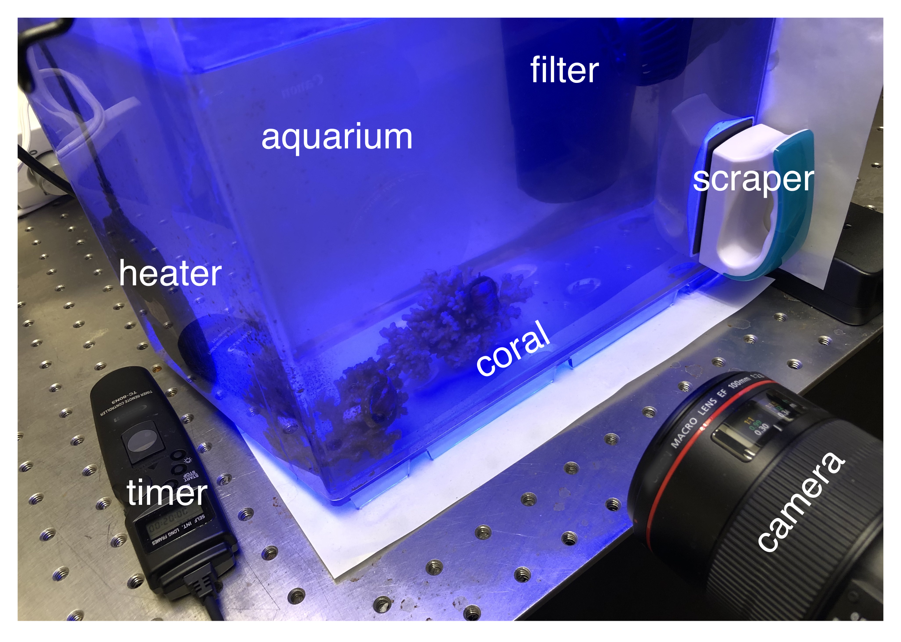


**Supplementary figure 1 | Experimental setup.** The experimental setup with a DSRL camera, a timer, an aquarium equipped with a filter, a scraper and a heater. The coral is fixed on another coral skeleton.


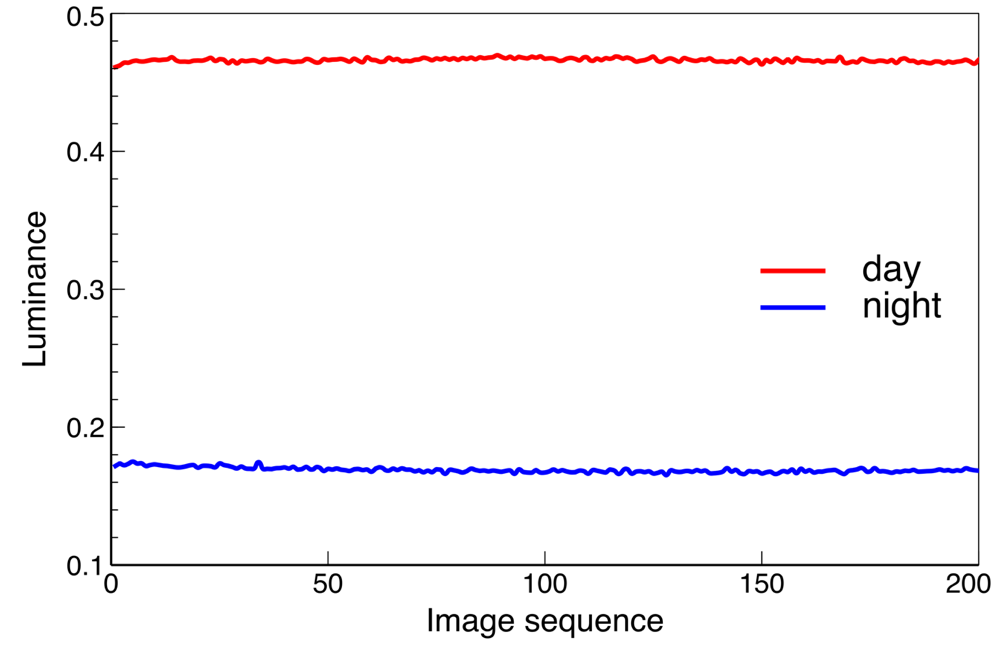


**Supplementary figure 2 | Luminance extracted from pictures.** Red and blue curves show the luminance at day and night, respectively.


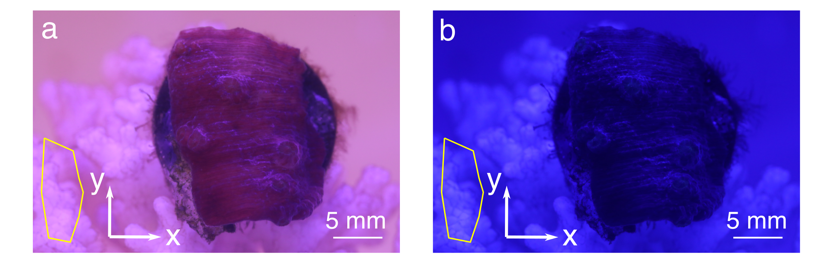


**Supplementary figure 3 | Pictures taken at day and at night.** The yellow curves in **a** and **b** enclose the coral skeleton in the aquarium. Scalebars are shown in **a** and **b**.


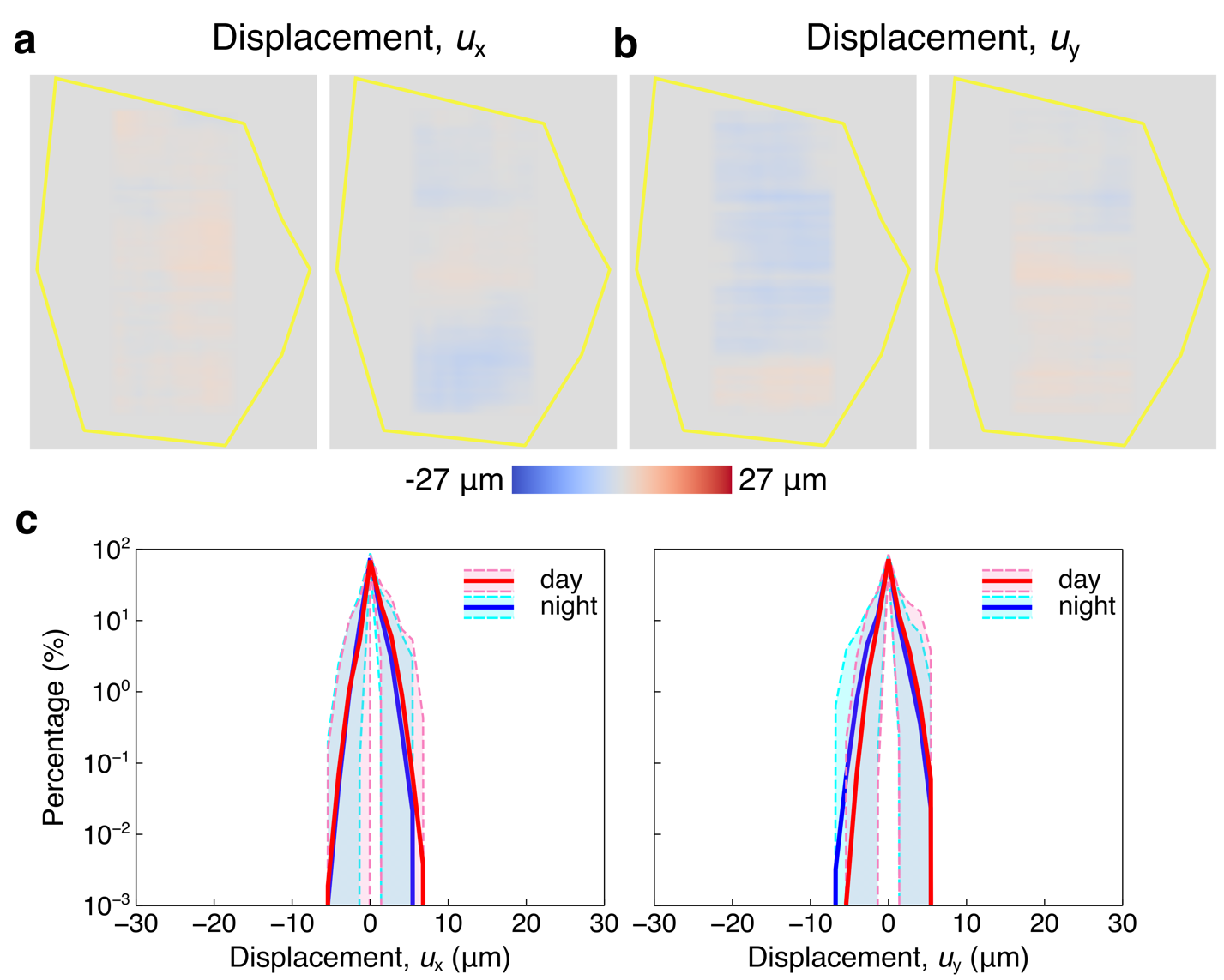


**Supplementary figure 4 | Displacements of the coral skeleton from digital image correlation.** **a.** The displacements $u_{x}$ along horizontal direction between the first and the second picture at day and night are shown in the left and right panel, respectively. **b.** The displacement $u_{y}$ along vertical direction between the first and the second picture at day and night are shown in the left and right panel, respectively. **c.** The percentage histograms of displacement $u_{x}$ and displacement $u_{y}$ are shown in the left and right panels, respectively. The red and blue curves indicate the means of each displacement at day and night. The pink and cyan regions are variation ranges at day and night.


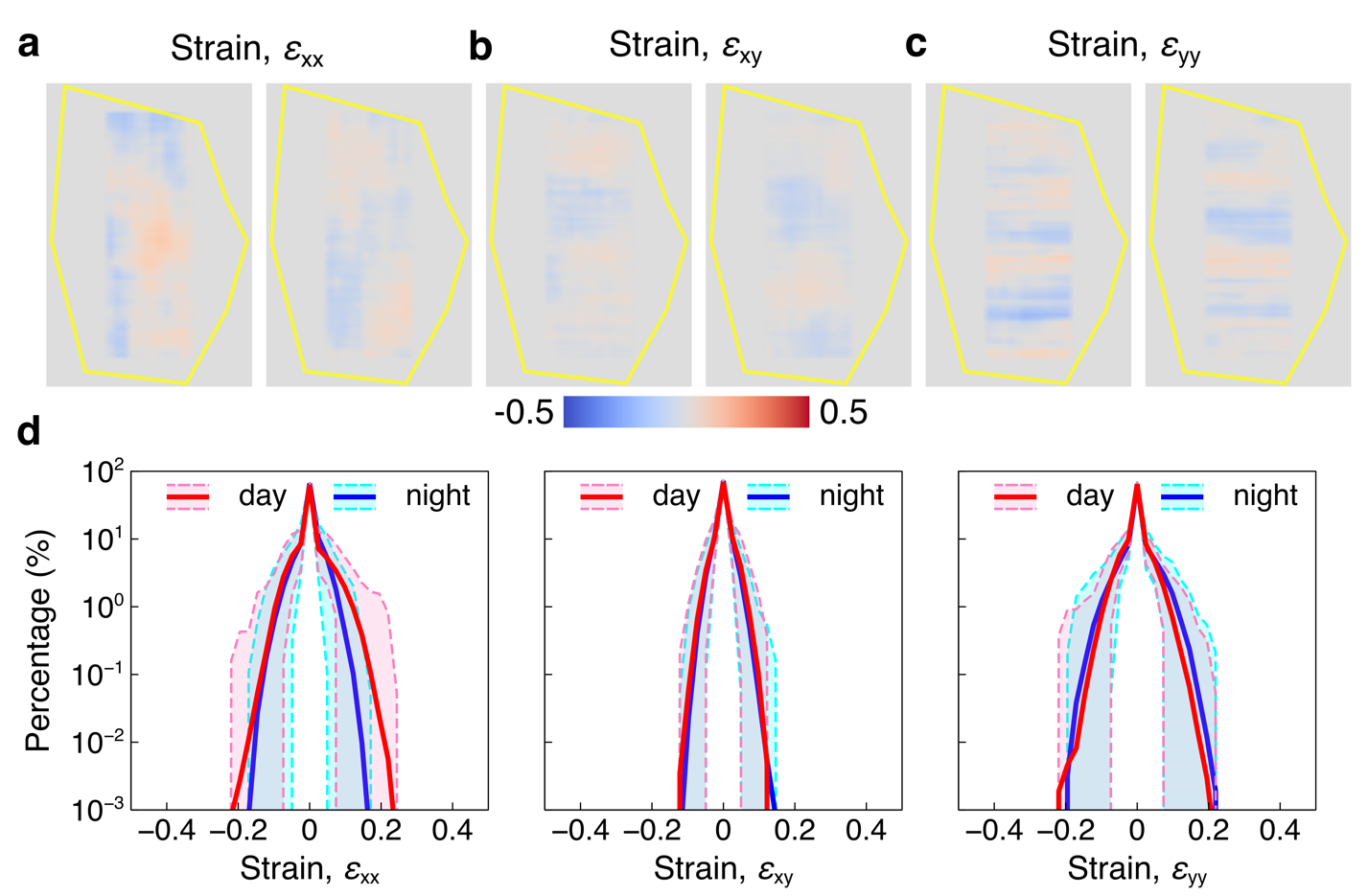


**Supplementary figure 5 | Strains of the coral skeleton from digital image correlation.** **a-c.** Strains $\varepsilon_{xx}$, $\varepsilon_{xy}$ and $\varepsilon_{yy}$ between the first and the second picture at day and night are shown in the left and right panel, respectively. **d.** The percentage histogram of the strains $\varepsilon_{xx}$, $\varepsilon_{xy}$ and $\varepsilon_{yy}$ are shown from left to right, respectively. The red and blue curves indicate the means of each strain at day and night. The pink and cyan regions are variation ranges at day and night.


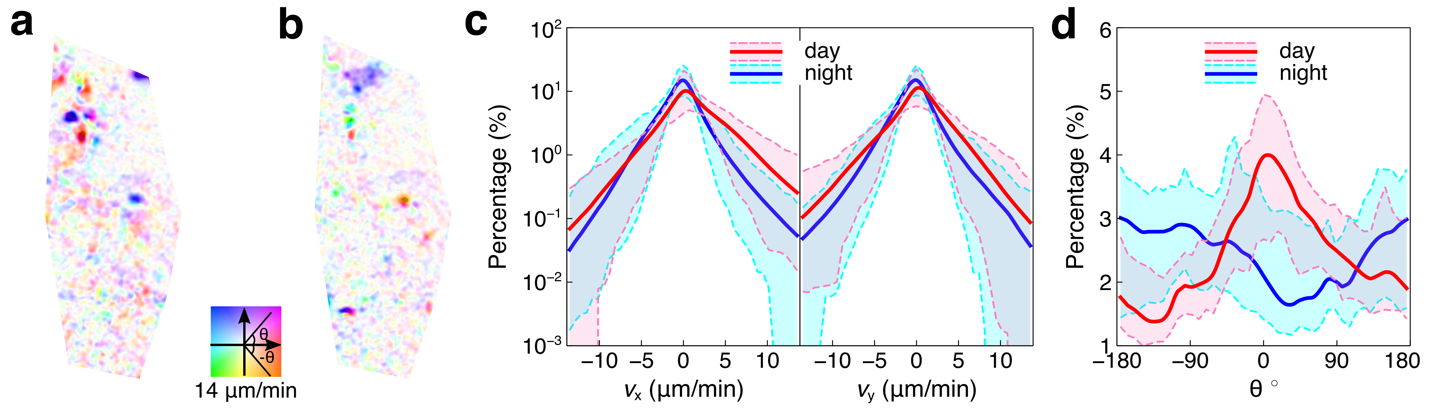


**Supplementary figure 6 | Optical flow of the coral skeleton.** **a-b.** Optical flow between the second and first pictures at day and night, respectively. **c.** The percentage histograms of velocities $v_{x}$ and $v_{y}$ along horizontal and vertical directions. The red and blue curves indicate the means of each velocity at day and night. The pink and cyan regions are variations at day and night. **d.** The percentage histogram of direction of velocity. The red and blue curves indicate the means of each angle at day and night. The pink and cyan regions are variation ranges at day and night.

**Supplementary movie**

Supplementary movie 1: Time-lapse video of *Montipora Capricornis* at day and at night

Supplementary movie 2: Displacement $u_{x}$ at day and at night

Supplementary movie 3: Displacement $u_{y}$ at day and at night

Supplementary movie 4: Histogram when taking 1^st^ picture as the reference picture

Supplementary movie 5: Strains at day and at night

Supplementary movie 6: Histograms when taking other pictures as the reference pictures

Supplementary movie 7: Optical flow at day and night

Supplementary movie 8: Modes at day and at night

Supplementary movie 9: Motion magnification in three frequency ranges at day

Supplementary movie 10: Motion magnification in three frequency ranges at night
